# Drawbacks and benefits associated with inter-organizational collaboration along the discovery-development-delivery continuum: a cancer research network case study

**DOI:** 10.1186/1748-5908-7-69

**Published:** 2012-07-25

**Authors:** Jenine K Harris, Keith G Provan, Kimberly J Johnson, Scott J Leischow

**Affiliations:** 1 George Warren Brown School of Social Work, Washington University in St. Louis, One Brookings Drive, Campus Box 1196, St. Louis, MO, 63130, USA; 2 Eller College of Management, McClelland Hall 405NN, University of Arizona, Tucson, AZ, 85721-0108, USA; 3 Mayo Clinic, 13400 E. Shea Blvd, MCCRB C-301, Scottsdale, AZ, 85259, USA

**Keywords:** Network analysis, Exponential random graph modeling, Cancer research, Translational research

## Abstract

**Background:**

The scientific process around cancer research begins with scientific discovery, followed by development of interventions, and finally delivery of needed interventions to people with cancer. Numerous studies have identified substantial gaps between discovery and delivery in health research. Team science has been identified as a possible solution for closing the discovery to delivery gap; however, little is known about effective ways of collaborating within teams and across organizations. The purpose of this study was to determine benefits and drawbacks associated with organizational collaboration across the discovery-development-delivery research continuum.

**Methods:**

Representatives of organizations working on cancer research across a state answered a survey about how they collaborated with other cancer research organizations in the state and what benefits and drawbacks they experienced while collaborating. We used exponential random graph modeling to determine the association between these benefits and drawbacks and the presence of a collaboration tie between any two network members.

**Results:**

Different drawbacks and benefits were associated with discovery, development, and delivery collaborations. The only consistent association across all three was with the drawback of difficulty due to geographic differences, which was negatively associated with collaboration, indicating that those organizations that had collaborated were less likely to perceive a barrier related to geography. The benefit, enhanced access to other knowledge, was positive and significant in the development and delivery networks, indicating that collaborating organizations viewed improved knowledge exchange as a benefit of collaboration. ‘Acquisition of additional funding or other resources’ and ‘development of new tools and methods’ were negatively significantly related to collaboration in these networks. So, although improved knowledge access was an outcome of collaboration, more tangible outcomes were not being realized. In the development network, those who collaborated were less likely to see ‘enhanced influence on treatment and policy’ and ‘greater quality or frequency of publications’ as benefits of collaboration.

**Conclusion:**

With the exception of the positive association between knowledge transfer and collaboration and the negative association between geography and collaboration, the significant relationships identified in this study all reflected challenges associated with inter-organizational collaboration. Understanding network structures and the perceived drawbacks and benefits associated with collaboration will allow researchers to build and funders to support successful collaborative teams and perhaps aid in closing the discovery to delivery gap.

## Background

The National Cancer Institute describes the scientific process around cancer research as a continuum beginning with scientific discovery, followed by development of interventions, and finally delivery of needed interventions to people with cancer. This discovery-development-delivery continuum conceptualization has been broadly adopted across healthcare and public health. In recent years, research identifying a major gap between discovery and delivery has accumulated [1-6]. One commonly cited study on the translation of medical discoveries into use in clinical settings found that the process of discovery to delivery takes an average of 17 years in the medical field [7]. In 2007, Chambers and Kerner [8] described the gap as follows: ‘Tested interventions are underutilized. Used interventions are under-tested.’ There has been some focus recently on scientific collaboration, or team science, as an important part of translational research that can help close the discovery to delivery gap [9-11]. Examples of this focus can be found in the text of recent funding announcements for delivery or implementation science projects at the National Institutes of Health (*e.g.*http://grants.nih.gov/grants/guide/pa-files/PAR-07-086.html).

Scientific collaboration, or team science, can involve researchers representing one or many fields from one or many organizations [12]. At its best, collaborative science incorporates diverse perspectives and stimulates new insights into complex problems and solutions. At its worst, conflicts based on longstanding scholarly disagreements hinder or halt progress [12]. Scientific collaboration relies on relationships, making social network analysis a uniquely useful tool in better understanding collaborative efforts [13,14].

While there has been a great deal of research over the past 20 years utilizing social network analysis [15], the few studies that have examined scientific collaboration using this perspective have focused on individual scientists. Examining connections among individuals is highly appropriate for trying to understand how ideas developed by one scientist or research team flow to another, enhancing the process of discovery and/or the process of moving ideas across the discovery-development-delivery continuum. However, this individual approach does not address how knowledge flows among organizations. This flow is important because organizations, such as research institutes or hospitals, often focus primarily on one stage in the discovery-development-delivery process, and may therefore represent narrow silos of knowledge. This is likely a major reason why the gap between discovery and delivery exists.

While networks of collaborative relationships that bring together diverse organizations across the discovery-development-delivery continuum would seem desirable, developing and maintaining such networks is fraught with challenges [16,17]. Essentially, participating organizations and those who work in them must recognize the advantages of collaborating with other organizations, whether within or across the discovery-development-delivery continuum, and must believe that the drawbacks to doing so are minimal. Building on previous research that described and compared the positions of individual organizations in the discovery, development, and delivery networks [18], in this study we focus on the relationships between organizations and examine the benefits and drawbacks associated with collaboration within each type of network. Specifically, we offer an explanation of the perceived benefits and drawbacks associated with collaborative relationships between organizations and how these benefits and drawbacks to collaboration might vary depending on the type (discovery, development, delivery) of cancer research being conducted. In doing so, we contribute to existing knowledge and approaches for understanding and evaluating collaborative science in two primary ways: we identify benefits and drawbacks associated with collaboration across the discovery-development-delivery continuum that will aid funders and researchers in strategically developing and managing research teams; and we demonstrate the use of exponential random graph modeling, which is a uniquely useful and accessible, yet underutilized, tool for understanding collaboration.

## Methods

Representatives of organizations involved in cancer research across Arizona that were represented on the research committee for the Arizona Cancer Control Program were invited to participate by completing a survey [18]. The Program is comprised of academic institutions, hospitals, the state health department, research institutes, and non-profit organizations involved in cancer research. The Program was formed by the Arizona Department of Health Services as part of a grant from the Centers for Disease Control and Prevention in 2003 to establish a comprehensive cancer control program. To identify members of the Program actively involved in cancer research, a working group comprised of Program research committee members reviewed research committee membership and identified 34 organizations. All 34 organizations were contacted; nine organizations indicated they were not involved in cancer research and several hospitals were found to be part of multi-hospital systems for which a single participant would be appropriate. After addressing these issues, the network consisted of 21 members. Most participating organizations named an administrative or medical director of cancer services or a director of research activities as the survey participant. Surveys were administered in 2007 with some follow-up to gather missing information in 2008. The survey response rate was 85.7% (18/21). The University of Arizona Institutional Review Board approved the study.

### Measures

In the survey, a list was provided of each of the organizations and the participant from each organization was asked to ‘please go through the list below and indicate with which organizations your organization/unit has been involved in its cancer control research for each type of research – discovery, development, and delivery.’ Operational examples of discovery, development, and delivery were provided along with examples of collaborative involvement:

1. Discovery: tissue sharing, basic scientific research, etc.

2. Development: synthesis of new knowledge, developing new treatments and drugs, developing new community programs, etc.

3. Delivery: research on delivery of treatment to patients, prevention and early detection, etc.

4. Collaborative involvement: shared personnel, shared information, joint programs, joint funding, shared facilities and equipment, support, advice, etc.

In addition, participants were asked to estimate the percent of time their organization’s cancer resources were spent on each of discovery, development, and delivery by responding to the following: Please estimate the percent of your organization’s/unit’s total cancer research resources that are spent on the following activities (note that the total should equal 100%): discovery (tissue sharing, basic research, etc.) ________%; development (synthesis of new knowledge, developing new treatment or drugs, etc.): _______ %; delivery (research on delivery of treatment to patients, research on prevention and detection, etc.): ______ %.

Finally, organizational representatives were asked to identify the benefits and drawbacks their organization had experienced during collaborations with other cancer research organizations. The list of benefits and drawbacks was developed based on earlier work by Provan *et al.*[19] combined with discussions with Program research committee members. For each drawback or benefit, respondents were asked to indicate whether the particular benefit and drawback ‘did not occur,’ ‘may have occurred,’ or ‘definitely occurred’ (Table 1).

**Table 1:** Percentage of organizations identifying each of 15 benefits and drawbacks of collaboration among organizations working on cancer research across Arizona

**Benefits**	**% (n)**
Acquisition of additional funding or other resources	88.9 (16)
Enhanced access to other knowledge	88.9 (16)
Enhanced reputation of my organization	83.3 (15)
Enhanced impact on other researchers	77.8 (14)
Enhanced influence on treatment and policy	72.2 (13)
Greater quality or frequency of publications	72.2 (13)
Improved access to study subjects or data	72.2 (13)
Development of new tools and methods	72.2 (13)
**Drawbacks**	
Frustration or aggravation in dealing with partners	72.2 (13)
Diversion of time and resources from other activities	61.1 (11)
Insufficient resources to support effective collaboration	61.1 (11)
Insufficient credit given to what my organization does	55.6 (10)
Difficulty due to geographical distances	50.0 (9)
Strained relations within my own organization	33.3 (6)
Loss of control/autonomy over decisions	27.8 (5)

### Data management

Three non-responders were dropped from analyses. Eighty to ninety percent of the pairs of network members, or dyads, in each of the three networks (discovery collaboration network, development collaboration network, and delivery collaboration network) agreed either that there was no collaboration (0) or there was collaboration (1) between them. However, a minority of dyads in each network consisted of members who disagreed (one member of the dyad indicated no collaboration while the other indicated collaboration). With 18 network members in an undirected network, there are 153 unique dyads (dyads = n*(n-1)/2). In the discovery network, 16 of the 153 dyads included a disagreement between the two network members in the dyad. In the development network, 30 of 153 dyads disagreed. In the delivery network, 27 of 153 dyads disagreed. Collaboration is inherently bi-directional (if I collaborate with you, you collaborate with me) so it was necessary to decide whether the dyads with disagreement would be coded as no collaboration or collaboration.

In previous work using these data, Provan *et al.* used confirmed ties only (*i.e.*, dyads where both members agreed there was a tie) [18]. Because the focus of that study was on explaining overall network structure, full agreement about the presence of every tie was an appropriate conservative approach for indicating who was connected to whom. For the analyses reported here, we decided to represent disagreements as collaborations. Methodologically, there are no specific coding rules for statistical analysis of networks regarding using confirmed ties or all ties [20], so the selection of a data management strategy is based on what is most appropriate for the study at hand. In this case, the recognition of a collaborative research tie between any two organizations might depend on whether the respondent him/herself is actually directly involved in, and thus, aware of, the collaboration. In addition, this study’s focus is on the relationship between dyadic ties and attitudes (regarding benefits and drawbacks) and not, as the previous study was, on whole network structure *per se*, suggesting the value of retaining all ties.

In addition to symmetrizing the network, we dichotomized the answers to the drawback and benefit questions. Participants selected from 0 (did not occur), 1 (may have occurred), and 2 (definitely occurred). Given the limited range of values and the large number of drawbacks/benefits, we dichotomized each to drawback or benefit to 0 (did not occur) and 1 (may have or definitely occurred). Phi correlations among the dichotomized benefits and drawbacks were examined to ensure that variables were capturing unique information. The benefits of publication and reputation were correlated at ϕ = 0.72 and the benefits of reputation and knowledge were correlated at ϕ = 0.79. The reputation benefit was therefore removed from analyses.

Finally, to examine whether there were important differences resulting from using all ties rather than confirmed ties, we conducted a sensitivity analysis comparing the patterns of benefits and drawbacks in networks with all ties and with confirmed ties only. This analysis revealed similar patterns across the networks resulting from these two data management strategies. The similarity was especially apparent for the discovery and delivery networks, which were the networks with fewer disagreements about ties in dyads.

### Analysis

We conducted descriptive, visual, and statistical analyses to examine the discovery, development, and delivery networks. Descriptive measures included network density, distribution of links per network member (degree distribution), and number of triangles in each network.

In addition to general network measures, we used an ERG-p* modeling (ERGM) strategy to develop statistical models predicting the likelihood of a tie between two network members based on characteristics of the network members, characteristics of their relationships, and general structural features of the network. In this type of modeling, the outcome is similar to logistic regression, but at the level of the dyad instead of the individual. That is, the model estimates the probability of a tie between any two network members; the sample size of interest, therefore, is the total number of dyads or possible ties in the network (n = 153). In the discovery network, a tie would indicate collaboration between two organizations on discovery research; in the development network, a tie indicated collaboration on development; and in the delivery network, a tie indicated collaboration on delivery of cancer research.

ERGMs were developed to examine the relationship between the drawbacks and benefits perceived by the organizations and the discovery, development, and delivery collaborations among these organizations. That is, we sought to determine which drawbacks and benefits were associated with increased or decreased likelihood of collaboration among cancer research organizations across the discovery-development-delivery continuum. Following Goodreau [21], ERGMs were developed in steps, starting with a null model, adding organizational characteristics, and, finally, adding terms designed to account for underlying global network structures.

The two global structures that are often found in observed networks, but are not well captured by statistical network models, are a non-uniform degree distribution and excess transitivity. A non-uniform degree distribution is the result of some network members having few connections, while other network members have many connections. The non-uniform degree distribution is often decreasing at a geometric rate in observed networks, with a large proportion of nodes being isolated or having very few connections and only a few nodes having many connections. This property of the degree distribution is captured by the geometrically weighted degree (GWD) term in statistical models of networks.

Transitivity is the relational quality that is expressed by the adage ‘the-friend-of-my-friend-is-my-friend’ and manifests in networks as triangle structures, where there is a link between A and B, a link between B and C, and a link between A and C, forming a triangle. Two terms account for transitivity in a network: the geometrically weighted edgewise shared partners (GWESP) accounts for partners shared by two connected nodes (a connected triangle), and the geometrically weighted dyadwise shared partners (GWDSP) accounts for partners shared by two nodes regardless of whether they are linked (a triangle with or without a base).

When models with all three geometric terms did not converge, we followed a strategy demonstrated by Hunter *et al.* using bivariate analyses to determine which of the geometric terms may be helpful in explaining existing global network structures [22,23]. The full model was then re-estimated retaining only those global terms that demonstrated a significant relationship with the outcome in this bivariate testing.

Model fit was examined at each step. Statistical measures of model fit, such as the Akaike Information Criterion (AIC) and Bayesian Information Criterion (BIC), may not be suitable for assessing statistical network models because network data do not meet necessary assumptions [22]. Therefore, graphic measures of fit based on examining a set of simulated networks were the primary tools used to select the final models. Specifically, the number of triangles in simulated networks based on each model was compared to the number of triangles in each model; similarly, we compared the observed degree distribution with the degree distribution for networks simulated based on the model. The models that produced simulated networks most closely approximating the observed network were selected as final models. Statistical network modeling was conducted using R-statnet.

## Results

The average number of benefits and drawbacks identified by each of the organizations was 9.06 (sd = 3.37), with a greater number of benefits (m = 5.44; sd = 2.01) than drawbacks (m = 3.61; sd = 1.91) to collaborating identified. The fewest organizations (n = 5) selected ‘loss of control/autonomy over decisions,’ while the most organizations (n = 16) identified ‘acquisition of additional funding or other resources’ and ‘enhanced access to other knowledge’ as characteristics of their collaborations. The proportion of organizations that selected each benefit and drawback is shown in Table 1.

**Figure 1:**
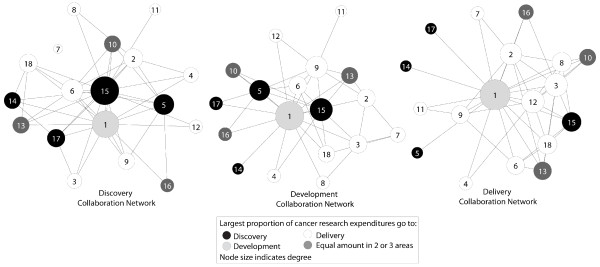
**Discovery, development, and delivery networks with nodes sized by degree centrality and colored by the area the organization spends the most time on related to cancer research**.

Organizations spent an average of 29.17% (sd = 28.87) of their cancer research resources on discovery research; 16.28% (sd = 16.36) on development; and 49.06% (sd = 33.21) on delivery. Table 2 shows the proportion of time on each activity for each of the 18 organizations. Four organizations reported no discovery research, six reported no development, and two reported no delivery. Two organizations reported spending 100% of their cancer research time on delivery research; all other organizations reported dividing their time between two or three of the discovery-development-delivery activities. Only one organization reported more resources going to development than to other activities. Figure 1 shows the three networks with organizations colored by the area where they spent the most cancer resources.

**Table 2:** The proportion of time spent by each organization on discovery, development, delivery research activities

**ID**	**% Discovery**	**% Development**	**% Delivery**
1	25	50	25
2	15	35	50
3	0	38	63
4	12	28	60
5	83	17	0
6	40	10	50
7	5	0	95
8	0	0	100
9	30	25	45
10	50	0	50
11	5	5	90
12	0	0	100
13	40	40	20
14	60	0	40
15	75	15	10
16	0	0	0
17	75	10	15
18	10	20	70

The nodes in Figure 1 are sized based on degree centrality; those with high degree centrality are well connected within the network. The one organization focused on development was most central in the development and delivery networks. In the delivery network, three of the four organizations focused on discovery were connected to the rest of the network only through their collaboration with the development-focused organization (Figure 1). In addition to being the most centralized, the delivery network was the densest network of the three indicating that more collaboration occurs around delivery than around discovery or development in this group of organizations. Table 3 shows average degree and other basic characteristics.

**Table 3:** Descriptive network statistics for the discovery, development, and delivery collaboration networks

	**Discovery**	**Development**	**Delivery**	**Average (sd)**
Density	0.29	0.26	0.30	0.28 (.02)
Degree (mean, sd)	9.78 (7.79)	8.89 (7.04)	10.22 (7.94)	9.63 (7.59)
Number of triangles	43	30	48	40.3

### Discovery model

**Figure 2:**
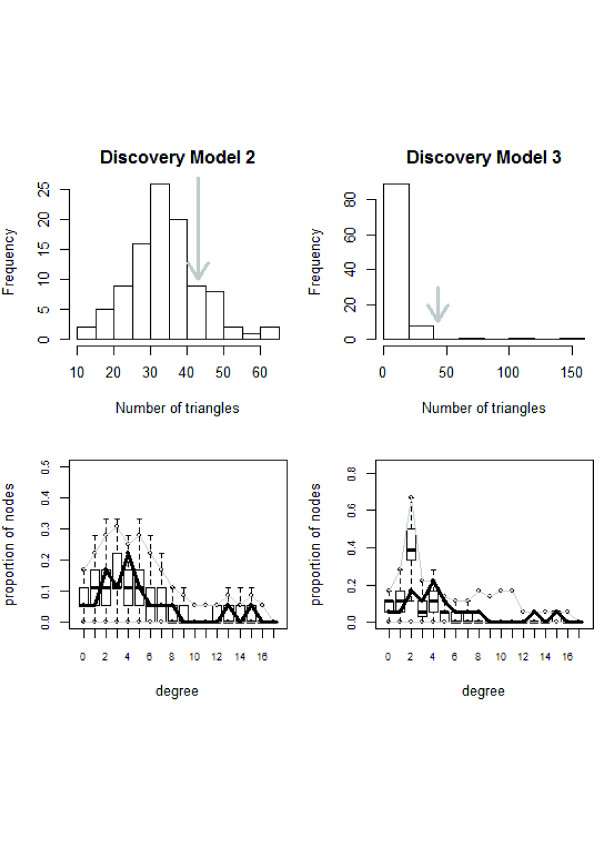
**Comparison of degree distribution and transitivity in simulations based on discovery model 2 (left) and discovery model 3 (right).** Gray arrows (top) and thick black lines (bottom) represent the observed network, while the histogram (top) and boxplots (bottom) show the simulated networks based on each model.

The full model including the three geometrically weighted terms did not converge. GWD and GWDSP were non-significant in bivariate analyses. These were removed and the full model was re-estimated. Goodness-of-fit plots (Figure 2) indicated that the characteristics-only model (Model 2) fit the data better than the full model (Model 3). Model 2 was selected as the final model for discovery (Table 4).

**Table 4:** Statistical network models of drawbacks and benefits associated with collaboration across the discovery-development-delivery continuum in a network of organizations working on cancer

	**(a) Discovery**			**(b) Development**			**(c) Delivery**		
	**Model 1 b (se)**	**Model 2 b (se)**	**Model 3 b (se)**	**Model 1 b (se)**	**Model 2 b (se)**	**Model 3 b (se)**	**Model 1 b (se)**	**Model 2 b (se)**	**Model 3 b (se)**
Edges	-.90 (.18)*	−5.71 (2.24)*	−8.44 (1.52)*	−1.04 (.18)*	−3.83 (1.79)*	−5.69 (1.41)*	-.84 (.18)	−3.84 (1.75)*	−2.57 (3.80)
Organizational characteristics (node attributes)									
% time discovery		0.06 (0.05)	.08 (.02)*		na	na		na	na
% time development		na	na		0.08 (0.03)*	.02 (.04)		na	na
% time delivery		na	na		na	na		−0.05 (0.03)	.51 (.06)*
Benefits									
Enhanced influence on treatment and policy		−3.03 (3.86)	−2.36 (.77)*		−5.11 (1.55)*	-.52 (.85)		−4.2 (2.21)	21.06 (8.85)*
Enhanced impact on other researchers		3.63 (2.07)	2.74 (.06)*		1.87 (1.45)	6.86 (.08)*		1.58 (1.68)	39.22 (.15)
Greater quality or frequency of publications		−1.95 (2.04)	−1.39 (.06)*		−1.69 (0.64)*	−1.61 (.08)*		−0.15 (1.23)	−22.56 (.17)*
Acquisition of additional funding or other resources		−5.07 (2.90)	−5.55 (.06)*		−5.63 (1.92)*	−3.21 (.10)*		−12.14 (3.45)*	24.88 (1.27)*
Improved access to study subjects or data		1.33 (1.37)	2.06 (.07)*		−1.15 (1.18)	.86 (.08)*		−1.31 (1.14)	20.43 (.14)*
Enhanced access to other knowledge		7.55 (6.09)	6.78 (.06)*		12.72 (4.03)*	−2.23 (.09)*		25.43 (7.5)*	−138.47 (.15)
Development of new tools and methods		−2.5 (1.64)	−2.44 (.07)*		−2.29 (1.14)*	.33 (.09)*		−4.61 (1.56)*	17.19 (.16)*
Drawbacks									
Diversion of time and resources from other activities		−1.1 (1.41)	−1.52 (.57)*		1.1 (0.69)	-.04 (.93)		−2.87 (0.81)*	.04 (2.71)
Insufficient resources to support effective collaboration		2.48 (1.73)	2.41 (.08)*		2.1 (1.11)	-.13 (.09)		4.78 (1.55)*	−8.30 (2.20)*
Loss of control/autonomy over decisions		3.15 (1.92)	2.82 (.49)*		2.76 (1.41)	-3.30 (1.07)*		9.48 (2.98)*	−58.55 (8.72)*	
Strained relations within my own organization		0.74 (2.05)	.93 (.14)*		−1.43 (1.13)	.13 (.66)		−1.75 (1.35)	-.35 (7.94)	
Frustration or aggravation in dealing with partners		1.35 (1.57)	1.99 (.62)*		−0.14 (1.14)	-.78 (1.21)		5.19 (1.52)*	−8.65 (8.19)	
Insufficient credit given to what my organization does		−2.02 (1.62)	−2.18 (.71)*		−1.38 (1.04)	1.57 (.70)		−9.16 (2.31)*	31.84 (4.60)*	
Difficulty due to geographical differences		−4.63 (1.20)*	−4.30 (.38)*		−3.98 (1.04)*	1.30 (.80)		−8.17 (2.28)*	44.04 (7.45)*	
Geometric terms (Global structural terms)										
Geometrically weighted degree (GWD)			na			na			−17.54 (2.38)*	
Geometrically weighted dyadwise shared partners (GWDSP)			na			1.10 (.10)*			17.49 (.42)	
Geometrically weighted edgewise shared partners (GWESP)			1.59 (.09)*			-.22 (.09)*			−5.45 (.22)	

*p<0.05.

In the final discovery model, only the drawback of geographical distance was significantly associated with discovery collaboration. The negative association between collaboration and distance indicates that organizations collaborating in this network were less likely to see geographical distances as a barrier. Table 3 shows the models for discovery, development, and delivery, with each final model indicated by the black box around it.

### Development model

**Figure 3:**
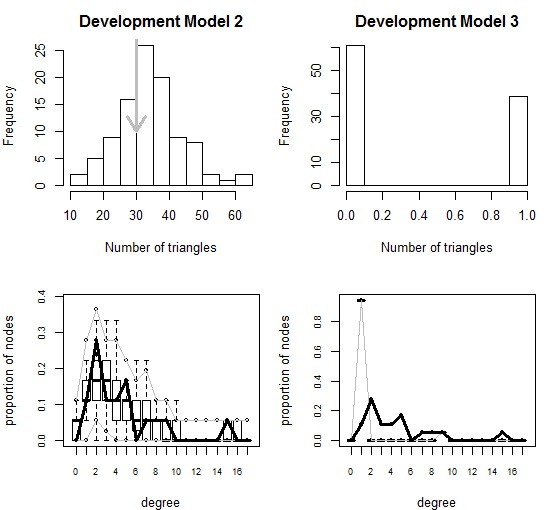
**Comparison of degree distribution and transitivity in simulations based on development model 2 (left) and development model 3 (right).** Gray arrows (top) and thick black lines (bottom) represent the observed network, while the histogram (top) and boxplots (bottom) show the simulated networks based on each model.

Like the discovery model, the full model for the development network did not converge with all three global terms. After testing, GWDSP and GWESP remained in the model. Goodness-of-fit plots were notably better for development Model 2 (Figure 3), so it was adopted as the final development model.

Several benefits and one drawback (geography) were significantly associated with collaboration around development. Organizations collaborating on development were less likely to perceive: ‘enhanced influence on treatment and policy,’ ‘greater quality or frequency of publications,’ ‘acquisition of additional funding or other resources,’ and ‘development of new tools and methods.’ However, collaborating organizations were more likely to report ‘enhanced access to other knowledge.’ Consistent with the discovery network, geography and collaboration were negatively associated; that is, organizations collaborating on development were less likely to find geographic distance as a barrier.

### Delivery model

**Figure 4:**
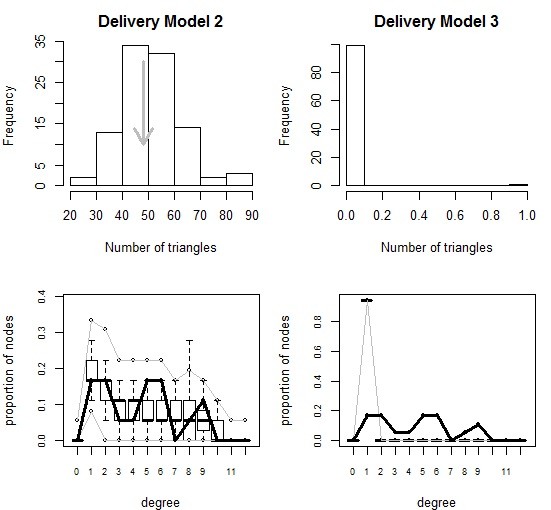
**Comparison of degree distribution and transitivity in simulations based on delivery model 2 (left) and delivery model 3 (right).** Gray arrows (top) and thick black lines (bottom) represent the observed network, while the histogram (top) and boxplots (bottom) show the simulated networks based on each model.

The delivery full model converged with all three geometric terms. However, based on goodness-of-fit plots, the full model was not a good fit and the characteristics only model was adopted (Table 3; Figure 4). Three benefits and six drawbacks were associated with delivery collaboration. Of the three benefits, only ‘enhanced access to other knowledge’ was positively related to a delivery collaboration. Significant drawbacks were equally divided between positive and negative association with delivery collaboration. Consistent with the other two networks, organizations collaborating on delivery were also less likely to identify geography as a barrier. The delivery model was the only model of the three to have multiple significant drawbacks associated with collaboration.

## Discussion and conclusions

Drawing from a social network perspective, we used network measures and statistical network modeling to examine the drawbacks and benefits associated with collaboration around discovery, development, and delivery in a network of organizations conducting cancer research. Examination of degree centrality in the networks demonstrated the important role of the one development-focused organization. Given the central role of development as the translator of research from discovery to delivery in the discovery-development-delivery continuum, the central position of the development-focused organization in the network makes intuitive sense, especially given the lack of additional organizations with a development focus. Public health research organizations might consider this important role and work to identify or cultivate partners with a focus on development or translational research.

The statistical network models identified a few similarities across the networks; however, in general, we found that different drawbacks and benefits were associated with each of the three stages in the discovery-development-delivery continuum. One consistent finding was that the drawback, ‘difficulty due to geographical differences,’ was negative and significant across all three networks indicating that collaborating organizations, regardless of the stage of research, were less likely to perceive geography as a barrier than organizations not collaborating. This finding is inconsistent with past research and may demonstrate a difference between perceptions and actual experience when it comes to long-distance collaboration. Specifically, a recent study on national tobacco control networks [24] found that the further apart organizations were geographically, the less likely they were to collaborate. It seems likely that these organizations perceived distance as a barrier, and therefore, did not pursue collaboration. Other studies have found that collaboration between multiple sites across distance is complex [25], despite the availability of numerous sophisticated communication technologies [26]. This complexity can lead to fewer project activities being conducted and fewer outcomes achieved [25]. In contrast, our findings indicate that once organizations are actually working together, geographical distance is no longer seen as a barrier.

Identification of geography as a barrier more closely related to perception than based on experience presents a challenge for the Centers for Disease Control and Prevention in their goal of developing Cancer Control coalitions in states. It may be necessary for additional effort to be placed on identifying and implementing ways to reduce the negative perception of geography as a barrier in order to encourage organizations that are more distant to make the effort to collaborate. For example, additional strategies such as the use of videoconferencing to foster increased communication may aid in addressing the problem. Such increased communication could also ameliorate other barriers.

In the delivery network, organizations that were collaborating were more likely to perceive ‘insufficient resources to support effective collaboration,’ ‘loss of control/autonomy over decisions,’ and ‘frustration or aggravation in dealing with partners’ as barriers to collaboration. Past research on team science in the delivery phase has stressed leadership and minimizing barriers; while this study does not address leadership, the existence of numerous barriers in the delivery network confirm this prior finding [27]. The perception of these barriers in the delivery network may signify the ‘growing pains’ that are likely to be encountered by organizations engaged in this emerging area of research. Specifically, organizations conducting delivery research may face challenges in bringing together stakeholders with varied expectations, goals, and incentives for collaboration. The positive association with insufficient resources, in particular, may be due to underestimating the resources needed to support the collaborations once they were initiated.

The National Cancer Institute (NCI) has identified, or re-framed, delivery science as implementation science (http://cancercontrol.cancer.gov/IS/definitions.html), which is defined as, ‘…the scientific study of methods to promote the integration of research findings and evidence-based interventions into healthcare policy and practice, and hence, to improve the quality and effectiveness of health services and care’ [28]. One of the goals of the NCI regarding implementation science is to build partnerships for the development, dissemination, and implementation of evidence-based measures, initiatives, and programs (http://cancercontrol.cancer.gov/IS/about.html). However, because research on collaborative science is also new and developing, there is limited information about how to build these partnerships in effective ways, building on benefits and minimizing drawbacks. While new funding mechanisms for implementation science are available (http://cancercontrol.cancer.gov/IS/index.html) and encourage transdisciplinary teams to apply, there is much that is not known about how the organizations that employ scientists interact. The findings reported here attempt to move this line of research forward. In addition, statistical network approaches like ERGM are becoming more accessible and widely used, providing a new way to examine how specific benefits and drawbacks, along with other characteristics, are associated with collaboration. As implementation science continues to develop at both the team and organizational levels, and as new tools and approaches are developed, like ERGM, to evaluate collaborative interactions, new information on how to structure effective teams and partnerships will likely emerge.

Three benefits were consistent in direction and significance across the development and delivery networks. The benefit, ‘enhanced access to other knowledge,’ was positive and significant in both networks indicating that organizations that were collaborating view improved knowledge exchange as a benefit of the collaboration. However, ‘acquisition of additional funding or other resources’ and ‘development of new tools and methods’ were both negative and significantly related to collaboration. So, although improved knowledge access is a likely outcome of collaboration, which is consistent with research in other fields [29,30], tangible outcomes of these collaborations including tools, methods, and funding are not being realized. These benefits may be more difficult to attain than others and may require more intensive collaboration over a longer period of time, possibly including the utilization of new approaches to communication to overcome barriers discussed above.

Additional significant benefits were identified in the Development network including negative associations between collaboration and the benefits of ‘enhanced influence on treatment and policy’ and ‘quality or frequency of publications.’ These may be serious challenges to collaboration at this level because the primary motivation of many researchers is to improve treatment or change policy. The rewards of developmental research may be more distal, with change occurring at the delivery step. At research-focused institutions and among junior faculty, the lack of direct influence on treatment and policy and the perception of reduced quality and frequency of publications is likely to dissuade collaboration on development projects. A few of the benefits were selected by nearly all of the participants, indicating consistent positive experiences following collaboration across the cancer research spectrum.

The findings of this study are limited in a few ways. First, the cross-sectional design does not allow examination of collaborations over time, thus limiting any understanding of how benefits and drawbacks emerge and change. Second, the limitation of examining a single coalition of organizations precludes generalizing findings to a larger population of cancer coalitions or other public health networks. Third, some of the variation in the identification of barriers and drawbacks was lost when these variables were dichotomized. Fourth, missing data prompted dropping three of the organizations in the study. We also recognize that other variables, such as readiness for collaboration [31], availability of collaboration technology [26], existence of collaborative leadership [32], and having supportive administrative infrastructures [31] might well provide additional understanding of when, why, and how collaboration across organizations takes place and whether it is successful or unsuccessful. Finally, despite the fact that use of a single key informant is common in studies of organizational networks, we recognize the shortcomings of this approach, especially for assessing benefits and drawbacks, which could reflect personal rather than organizational views. Despite these limitations, our study is the first we are aware of to examine the benefits and drawbacks associated with research collaboration along the discovery-development-delivery continuum. Thus, our work contributes to a deeper understanding of implementation science, especially as it occurs across organizations.

Given the importance of understanding how research moves along the discovery-development-delivery continuum, and the role of collaborative efforts in advancing science, it will be important for future studies to examine these and other drawbacks and benefits associated with collaboration at each phase of the research continuum. It is of particular importance, also, to gain understanding about how the benefits and drawbacks influence the flow of knowledge from discovery to delivery. For example, once a research team has made a discovery, what are the specific benefits and drawbacks to continuing to work as a team to translate the discovery from the lab to the clinic, and to what extent do the organizations that employ the scientists either facilitate or hinder successful interactions? In addition, information about how drawbacks might be mitigated or benefits might be strengthened is needed. Further investigation of these and related issues would be especially helpful to those who are involved in the research process, and also to health policy officials and funders in developing incentives for researchers in cancer and other areas to collaborate across organizations and across the research continuum.

For funders in particular, understanding what scientists perceive as the benefits and challenges of collaborative research is important for shaping research initiatives, which not only encourage, but often require, collaboration. Based on our findings, scientists recognized that collaboration led to benefits like enhanced access to knowledge and breaking down perceived barriers due to geographical distance. However, we also found some benefits, like ‘acquisition of additional funding or other resources,’ were not perceived as likely outcomes of collaboration, while others, such as ‘enhanced influence on treatment and policy,’ were negatively related to collaboration. Funding agencies may wish to evaluate funded projects to identify strategies used by collaborative research teams that were successful in acquiring additional funding, improving treatment or policy, and other areas. Funders could then provide guidance and specific examples of the benefits of collaborative research in order to help scientists recognize its potential. As evidence about the benefits and drawbacks of collaborative science accumulates, funders may wish to adapt the collaboration requirements of research initiatives accordingly.

## Abbreviations

ERGM, Exponential random graph modeling; GWD, Geometrically weighted degree distribution; GWDSP, Geometrically weighted dyadwise shared partners; GWESP, Geometrically weighted edgewise shared partners.

## Competing interests

The authors declare that they have no competing interests.

## Authors’ contributions

JH led the manuscript development and conducted all analyses. KP aided in the initial project conceptualization and led the data collection process. KP also aided in the overall development of the manuscript, writing of manuscript sections, and editing the final draft. KJ aided in writing sections of the manuscript and editing the final version of the manuscript. SL led the initial project conceptualization and helped guide the data collection. SL also aided in writing of sections of the manuscript and editing the final draft. All authors read and approved the final manuscript.
